# Effects of Intensive Fattening With Total Mixed Rations on Carcass Characteristics, Meat Quality, and Meat Chemical Composition of Yak and Mechanism Based on Serum and Transcriptomic Profiles

**DOI:** 10.3389/fvets.2020.599418

**Published:** 2021-01-21

**Authors:** Yi-Xuan Liu, Xiao-Ming Ma, Lin Xiong, Xiao-Yun Wu, Chun-Nian Liang, Peng-Jia Bao, Qun-Li Yu, Ping Yan

**Affiliations:** ^1^College of Food Science and Engineering, Gansu Agricultural University, Lanzhou, China; ^2^Animal Science Department, Lanzhou Institute of Husbandry and Pharmaceutical Sciences, Chinese Academy of Agricultural Sciences, Lanzhou, China; ^3^Key Laboratory for Yak Genetics, Breeding, and Reproduction Engineering of Gansu Province, Lanzhou, China

**Keywords:** carcass characteristics, intensive fattening, meat quality, nutritional value, total mixed rations (TMR), yak

## Abstract

The objective of this study was to investigate the effects of intensive fattening with total mixed rations (TMR) on carcass characteristics, meat quality, and chemical composition of the yak meat. Theoretical data has been provided for evaluating the quality of yak meat during natural grazing and short-term fattening. Based on the analysis, we found that in fattening yak, the carcass weight (CWT) was increased by 106.43%, whereas the cooking loss, tenderness, and drop loss were significantly improved due to higher intramuscular fat content and lower moisture (*P* < 0.05). Protein, fat, calcium, and amino acids were also much higher (*P* < 0.01) in fattening yak compared with the grazing yak. The levels of albumin (ALB), lactate dehydrogenase (LDH), triglyceride (TRIG), and amylase (AMYL) in serum indicated better nutritional status for fattening yaks. The transcriptomics analysis showed that the high expression of ACSL1 and ACACB genes improved the synthesis and deposition of fat in fattening yak, whereas the regulation of SLC7A8, ATP1A4, ATP1A1, SLC3A2, and CPA3 gene expression weakened the proteolysis. These results indicated that fattening with TMR improves the yield and quality of the yak meat.

## Introduction

The yak (Poephagus grunniens or Bos grunniens) is a scarce and precious farm animal that lives in the Qinghai–Tibetan Plateau and surrounding regions of China (1). There are ~20,000,000 yaks globally, and the yield of yak meat is ~300,000 tons per year. Yak was domesticated by humans between 7,000 and 10,000 years ago, and has since migrated along with the Tibetan population across the plateau, providing with meat, milk, transportation, shelter, and fuel to the residents (2). Yak meat is the primary source of animal protein in the human diet under low temperature and oxygen deficits (3). This cold environment-adapted animal produces meat with a composition of low and favorable fat and a high protein with mineral content, satisfying the discerning meat consumer (4). Besides, it contains several active functional components, such as eicosapentaenoic acid (EPA), docosahexenoic acid (DHA), and conjugated linoleic acid (CLA), etc. (5). Hence, yak meat has become increasingly popular among consumers beyond the Tibetan plateau in recent years (6).

In Qinghai-Tibetan Plateau, the four seasons are not definite due to a high altitude and topography. Natural grazing is the traditional breeding mode of yak; compared with relatively high forage quality in warm season, half a year of the withering of grass period in cold season affects its nutritional level and growth. However, several factors affect the production of yak. In the traditional mode of breeding, yak meat is supplied seasonally with a long production cycle, unstable quality, and low yield, leading to unsuitable growth status for slaughter and thereby, the supply of fresh yak meat is stopped in the cold season (1). Therefore, it is crucial to develop a different management system from conventional yak production and upgrade the sustainability of the agricultural output in the Qinghai-Tibet Plateau and its adjacent regions.

Nutrient intake was considered one of the essential factors in determining the meat quality of animals (7). When the intake of nutrients exceeds the expenditure for maintaining life activities, animal's weight begins to increase. Blood biochemical indicators reflect the nutrient metabolism in animals, and it can also represent the growth (8). Besides, the nutritional ingredient in muscles is highly associated with meat quality (9).

Various modern animal management methods have been developed to improve growth efficiency and meat quality, including intensive fattening (10). However, when adequate nutrition is provided to yak by an intensive fattening system during the cold season, the efficiency of the production system is improved, and the final meat product is increased by satisfying the demand of the customer (11).

Total mixed ration (TMR) provides forage and simultaneously increased the digestibility of nutrients and digestible energy content (12). It has several advantages, providing homogeneous feed to animals over time, reducing feed costs, and saving time (13). However, intensive fattening is better than other farming systems, regardless of age and sex (14). Hence, it has been widely used to feed animals, including bull, heifer and goat (15–17).

Fattening technology was generally used in animals for feeding and breeding research, as observed in the previous reports. However, few studies on the use of TMR in yak breeding has been reported. We speculated that the fattening yak with TMR might promote muscle growth, boost the efficiency of yak's yield and meat quality.

The yak might have different biological capabilities to adapt to the availability of feeding, so the yak intensive fattening mechanism used by TMR may vary from other animals. Previously, transcriptomic studies have been applied to analyze differentially expressed genes (DEGs) (4) and forecast the relationship between genotypes and phenotypes (18). In recent years, high-throughput RNA sequencing (RNA-seq) is developed, offering several advantages over other transcriptome profiling methods such as microarray analysis or real-time PCR. It has been used successfully to study muscle growth of cattle, yak and pig (18–20).

Researchers have done several analyses, focusing on different breeds, ages, and supplementary feeding methods. The present research focuses on the cold season, which significantly affects traditional grazing feeding methods, and short-term intensive fattening of yaks, using transcriptomics and molecular biotechnology based on theory and experiments to identify related genes yak muscle development. Further, the response mechanism of yak meat quality was determined to the nutritional regulation of intensive fattening with TMR. Adult yaks gain higher feed energy by depositing fat from short-term fattening. When fat is stored to a certain extent, it will suppress its appetite, transform feed continuously, and release it in time to prevent food waste. We have provided theoretical data for promoting the development of the yak industry and nutritional regulation of short-term fattening in yak's meat quality, making it suitable for developing animal husbandry in the cold season at high altitude and cold pasturing areas.

## Materials and Methods

### Evaluation of Animals and Feeding Systems

The animal studies and procedures were approved by the Ethics Committee of the Gansu Agricultural University and the Ethics Committee of Lanzhou Institute of Husbandry and Pharmaceutical Sciences, Chinese Academy of Agricultural Sciences (2019-002). The fattening mode of the yak was carried out by Xiahua Halal Food Co., Ltd. in Haiyan County, Haibei Prefecture, Qinghai Province. There are three phases in a year, with 1,000–1,200 yaks per phase. The yak selected in this experiment is the third phase (November ~ next March). In order to avoid the company's production, a total of 12 male yaks (5 years old) with similar body weight (270 ± 10 kg) were selected during the experiment, which was randomly divided into grazing group (*n* = 6, weight 268 ± 7.3 Kg) and fattening group (*n* = 6, weight 271 ± 5.2 Kg). After being stirred by a horizontal TMR mixer, the staff drove in a small tractor to manually spread the material, feeding twice a day where fattening yaks could forage TMR and drink water from the water trough *ad libitum*. Simultaneously, the grazing system is carried out, wherein yaks in the grazing group could also eat forage *ad libitum* without any supplementary feeding. Besides, the live weight of each group was measured before slaughter.

It also needs to be added that a professional meat processing company cultivates the selected animals (5-year-old male yaks) to improve meat production performance. Reproduction occurs when they get matured, and thereafter no other work is done. They are slaughtered in adulthood following conventional practices. Secondly, the perspective of nutritional regulation of yak meat quality without considering its economic benefits of animal feeding has been described in this paper.

### Introduction of Sampling Site and Experimental Diet

The geographical coordinates are 100°23' to 101°20'E and 36°44' to 37°39' N in a pasture in Haiyan County, Haibei Prefecture, Qinghai Province. The cold period is long in the site, and the cooling period is short, sunshine is sufficient with intense solar radiation, dry and wet seasons are distinct, rain and heat are in the same season with sufficient night rains and strong winds. This area belongs to a typical alpine landform of plateau continental climate. The annual average temperature is −2.4 °C ~ 1.4°C, with the highest and lowest temperature is 33.3°C and −36.3°C, respectively. The annual average precipitation is 309.9 ~ 529.1 mm, whereas the annual sunshine hours are 2517.6 ~ 2995.3 h. However, the average annual radiation dose is 5210.2 MJ m^−2^ ~ 6568.3 MJ m^−2^. There is no absolute frost-free period as most areas are above 3,000 m from the sea level. There are 3.625 million mu (about 242,000 hm^2^) of grassland in summer and 1.532 million mu (about 102,000 hm^2^) of grassland in autumn. In the experiment, *Kobresia Pygmaea* was used as the dominant species, whereas the associated species were *Poa annua, K. myosur-oides, Stipa purpurea, S. alien-Na, Achnatherum Splendens, Po-lygonum vivparum, Cremanthodium linrare*, etc. In spring (April), and randomly select ten 1 m^2^ meadow in the pasture, each of which was about 0.5 kg. The forage was stored at 4°C in a sealed bag. After being dried naturally in the laboratory, the forage was crushed and ground fine with a feed mill. Local fattening farms provide TMR samples, and the processing methods are the same as forage.

Table 1 shows the composition of nutrients of the forage and TMR diet. Based on the Chinese beef cattle breeding standards (2004), beef cattle with a body mass of 270 kg, average daily gain (ADG) of 1,000 g/d, needs various nutrition, such as maize, soybean meal, wheat bran, and flaxseed. It is used as a raw material for the supplementary diet, such as hay, dried distillers grains as roughage, resulting in a fine to coarse ratio was 3:7. The nutritional level of TMR is much higher than the forage. It was reported that a high protein diet may significantly improve animal performance (21).

**Table 1:** Composition and nutrient levels of diets (DM basis).

**Project**	**Content**	
Raw material composition, %		
Maize (Zea mays L.)	60.00	
Soybean meal	10.00	
Distillers dried grains with solubles (DDGS)	6.00	
Flaxseed meal	10.00	
Wheat bran	2.00	
Salt	1.00	
Premix ①	2.00	
Stone powder	1.00	
Sodium bicarbonate	1.50	
Yeast powder	6.50	
Total	100.00	
Nutrient content, %②	Forage	TMR
Dry matter	91.03	94.25
Crude protein	7.40	14.62
Neutral detergent fiber	61.05	33.06
Acid detergent fiber	40.54	24.44
Crude fat	0.89	4.34
Coarse ash	8.97	6.74
Calcium	3.73	0.44
Phosphorus	0.031	0.37

*^①^Premix provides 1 500 IU of vitamin A, 550 IU of vitamin D, 10 IU of vitamin E, 20 mg of iron, 40 mg of manganese, 30 mg of zinc, 0.50 mg of iodine, 0.30 mg of selenium, 0.2 mg of cobalt per kg of diet. ^②^Other nutritional indicators are measured values*.

### Slaughter Procedure and Samples Collection

At the end of the feeding experiment, all yaks were fasting between 08:00 a.m., and 10:00 a.m. before slaughter, and 20 mL blood samples were collected from the jugular vein of each yak and centrifuged at 3,000 × g for 15 min at 4°C using a KL05R refrigerated centrifuge (Kaida Inc., Changsha, China). Serum samples were collected and stored at −20°C for the analysis of biochemical parameters. After that, the weight of all yaks has been measured. All yaks were slaughtered in Qinghai Province following the Chinese guidelines for using experimental animals and animal welfare, approved by the Gansu Agricultural University and the Lanzhou Institute of Husbandry and Pharmaceutical Sciences, Chinese Academy of Agricultural Sciences. Twenty minutes after slaughter, the carcass weight was recorded. The LD (12th-13th rib level) of each yak were quickly collected and divided into two parts in which a larger piece had about 1,000 g while the smaller one had about 10 g. A small piece of muscle sample was cut into 1 cm × 1 cm cubes and immediately put in enzyme-free cryopreservation tubes and liquid nitrogen for molecular biology experiments. The two large pieces of muscle samples were placed separately in two sealed pockets. One was used to measure color, pH, shear force, cooking percentage, and drop loss, while the other was frozen at −20°C for analysis of the chemical composition of meat. The carcasses of yaks were boned and then weighted the bones and meat of each yak.

### Meat Quality Measures

The meat quality was analyzed using the methods described by the Association of Official Analytical Chemists (AOAC, 2015). CIE LAB color space was used for determining the meat color (CIE, 1976). Meat color was measured at 45 min and 24 h postmortem at 4°C by CR-400 chroma meter (KONICA MINOLTA Inc., Tokyo, Japan). For each muscle sample, three measurements were taken, and the results were expressed as Lightness (L*), redness (a*), and yellowness (b*). pH was measured using a TESTO 205 pH meter (TESTO AG Inc., Lenzkirch, Germany) at 45 min and 24 h of postmortem. C-LM4 tenderness meter (Northeast Agricultural University, Shenyang, China) was used to measure shear force, whereas DDSY-318 EC meter (Leici Inc., Shanghai, China) was used to measure electrical conductivity by inserting the instrument into the center of LD perpendicular to the muscle fiber direction at three different positions. For determining the drop loss, trimming the fat and connective tissue around the meat after slaughter, the specimens were cut into a dimension of 6 cm × 3 cm × 2 cm. The specimens were then hanged using disposable paper cups, sealed the surfaces with plastic wrap to ensure that they were in contact with the glass. Then, the samples were kept at 4°C in a refrigerator. The percentage of drop loss can be calculated as follows:

Drop loss (%) = (*D*_1_ − *D*_2_)/*D*_1_ × 100

The percentage of cooking loss can be represented as follows:

Cooking loss (%) = (*C*_1_ − *C*_2_)/*C*_1_ × 100

### Nutritional and Chemical Components Analysis of Meat

The contents of moisture, protein, fat, ash, calcium, and phosphorus in the LD of yaks have been analyzed following the AOAC procedure (AOAC, 2015). Moisture in the LD of yaks was determined by drying at 105°C overnight in a 101–4 thermoelectric thermostat drying box (Xinyi Inc., Shanghai, China). Ash in the LD of yaks was determined by heating at 550°C in a KSW-12-12 muffle furnace (Yunxin Inc., Beijing, China). Crude protein in the LD of yaks was measured by the Kjeldahl method with 8400 KelTec system (FOSSInc., Hillerød, Denmark), as referred by the national standard (GB/T 6432-2008). Fat in LD of yaks was extracted in a Soxtec 2050 soxhlet apparatus (FOSSInc., Hillerød, Denmark) using petroleum ether, whereas the calcium was determined by EDTA titration method. Besides, phosphorus was determined by vanadium-molybdenum-yellow-spectrophotometry with UV-2550 ultraviolet and visible spectrophotometer (SHMIADZU Inc., Kyoto, Japan).

The amino acids were quantified based on Chinese recommended standards (GB 5009 124-2016). The protein was broken up into free amino acids by acid hydrolysis at 105°C for 22 h, and they were separated by ion-exchange column separation and derived with a ninhydrin solution. The derivatives were detected using the automatic amino acid analyzer (Biochrom 30+, Biochrom, UK). The flow rate of eluent was 1 mL/min, while the injection volume was 10 μL. The absorbance wavelength was found to be 254 nm. The temperature of the column was controlled at 40°C. The content of the derivatives was determined under the above conditions, and the amino acid content was calculated based on the derivative.

The fatty acids in the LD of yaks have been detected based on the method described in (22). The LD was extracted by the mixed solution of chloroform and methanol (v:v, 2:1), and the obtained solution was then dried under a nitrogen blow to get fat. Next, 10 mg of yak-fat samples were placed in a centrifugal tube and decomposed into fatty acids by hydrolysis using 2 mL of 0.1 M sodium hydroxides methanol solution. Two ml of boron fluoride-methanol solution was then added to derive the fatty acids. At last, 2 mL n-hexane was used to extract the fatty acid methyl esters (FAMEs). The gas chromatography system (7890A, Agilent Corp., Santa Clara, US) coupled with a flame ionization detector (FID) was used to analyze the content of FAMEs. Agilent J&WCP-Sil88FA- ME capillary column (100 × 0.25 mm × 0.20 μm) was chosen to separate the FAMEs. Finally, analytes were determined based on their retention times, and fatty acids were calculated by FAMEs.

### Serum Sampling Analysis

The glucose (GLU), creatinine (CREA), albumin (ALB), alanine transaminase (ALT), triglyceride (TRIG), amylase (AMYL), lipase (LIPA), creatine phosphokinase (CK), lactate (LAC), lactate dehydrogenase (LDH), alkaline phosphatase (ALKP) and cholesterol (CHOL) in serum sample of yaks were analyzed using an automatic biochemistry analyzer (IDEXX Catalyst One, US).

### Transcriptome Analysis

Total RNA was isolated by trizol reagent from the LD of yaks (Invitrogen, Carlsbad, CA). There were 12 samples, and their OD260/OD280 = 1.93. RNA purity was checked using the NanoPhotometer® spectrophotometer (IMPLEN, CA, USA). RNA integrity was assessed using the RNA Nano 6000 Assay Kit of the Bioanalyzer 2100 system (Agilent Technologies, CA, USA).

Sequencing libraries were generated using NEBNext® UltraTM RNA Library Prep Kit for Illumina® (NEB, USA), as recommended by the manufacturer, and index codes were added to attribute sequences to each sample. For selecting cDNA fragments of preferably 250–300 bp in length, the library fragments were purified with the AMPure XP system (Beckman Coulter, Beverly, USA). PCR was performed with Phusion High-Fidelity DNA polymerase, Universal PCR primers, and Index (X) Primer. Finally, PCR products were purified (AMPure XP system), and library quality was assessed using the Agilent Bioanalyzer 2100 system.

Clustering of the index-coded samples was performed on a cBot Cluster Generation System using the TruSeq PE Cluster Kit v3-cBot-HS (Illumia), as instructed by the manufacturer. After cluster generation, the library was sequenced on an Illumina Novaseq platform, and 150 bp paired-end readings were generated.

### Statistical Analysis

Data obtained from carcass characteristics, meat quality, meat chemical composition, and blood biochemical index were analyzed using the SPSS package (SPSS 23.0, Chicago, IL, USA) to determine the significant difference. The least-squares mean (LSM) was separated using an independent-sample *t*-test. The standard error of the mean (SEM) was obtained as the standard deviation (SD) divided by the square root of the sample size. Each experiment was performed three times. The results were presented as mean values ± standard deviation (SD). *P* < 0.05 was used as a criterion for significant difference and *P* < 0.01 as the criterion for an extremely significant difference.

Raw transcriptomics data in FastQC format was first processed through in-house Perl scripts. All the downstream analyses are based on high-quality clean data. The index of reference genome was developed using Hisat2 v2.0.5, while paired-end clean reads were aligned with the reference genome using Hisat2 v2.0.5. We selected Hisat2 as a mapping tool to allow Hisat2 and generate a database of splice junctions based on a gene model annotation file, thus obtaining a better mapping result than other non-splice mapping tools. The expression level of each transcript was calculated based on the fragments per kilobase of exon model per million mapped reads (FRKM). Differentially expressed genes (DEGs) between the feed mode have been identified using the statistical significance of the absolute value of |log2 fold change| > 1 and false discovery rate (FDR) <0.01. GO enrichment analysis and KEGG pathways have been implemented by the cluster Profiler R package, in which gene length bias was corrected.

## Results

### Carcass Characteristics and Meat Quality

Table 2 shows the average weight of the two groups of yak before starting the experiment, including the final weight (live weight before slaughter) and average daily gain. The final weight of yaks was varied significantly between the grazing yak and fattening yak (240 vs. 400 kg, *P* < 0.05). Even the CWT of a fattening yak was significantly higher than that of grazing yak (230.17 vs. 111.50 kg). In the fattening group, the dressing percentage was 57.52%, which was considerably higher than that in the grazing group (46.45%) (*P* < 0.01). Meat percentage of yak in the fattening group was significantly higher (46.25%) than the 32.20% found in the grazing group (*P* < 0.01). The meat-bone ratio of yak in the fattening group was 5.37, as higher than 2.93 in the grazing group (*P* < 0.01).

**Table 2:** Effects of intensive fattening with TMR on the carcass characteristics of yak (mean ± SD).

**Carcass characteristics**	**Grazing yak**	**Fattening yak**	***P***
Initial weight (kg)	268.00 ± 7.30	271.00 ± 5.20	0.476
Final weight (kg)	240.17 ± 7.86^A^	400.00 ± 6.73^B^	<0.01
Average daily gain (kg)	−0.17 ± 0.04①^A^	0.87 ± 0.06^B^	<0.01
Carcass weight (kg)	111.50 ± 8.51^A^	230.17 ± 9.19^B^	<0.01
Dressing percentage (%)	46.45 ± 0.96^A^	57.52 ± 0.55^B^	<0.01
Meat percentage (%)	32.20 ± 0.99^A^	46.25 ± 0.68^B^	<0.01
Meat-bone radio	2.93 ± 0.70^A^	5.37 ± 0.13^B^	<0.01

*^①^The average daily gain is calculated and the average daily gain in grazing yak is negative. Different lowercase letters a, b in the same peer indicate P < 0.05, capital letters A, B indicate P < 0.01 (the same below)* .

Table 3 presents the meat quality of the LD of yaks in two feeding modes. Significant differences in meat color were observed at 45 min postmortem between the grazing group and the fattening group (*P* < 0.01). There were no differences in L^*^ and b^*^ at 24 h postmortem between two groups (*P* > 0.05); however, a^*^ at 24 h postmortem in the fattening group was lower than that in the grazing group (*P* < 0.05). The pH at 45 min and 24 h did not show any significant differences (*P* > 0.05) between the two groups. Shear force showed significant differences (*P* < 0.05) between the fattening yak muscle and grazing yak muscle (8.76 N vs. 10.88 N). Intensive fattening with TMR increased (*P* < 0.05) the cooking loss (37.21% vs. 29.93%) while decreased (*P* < 0.05) the drop loss (20.36% vs. 18.11%) of yak muscle. At the same time, there was no significant difference (*P* > 0.05) in conductivity between the two groups.

**Table 3:** Effects of the intensive fattening with TMR on meat quality in *longissimus dorsi muscle* of yaks ($\bar{\text{x}}\pm$ s, %).

**Meat quality**	**Grazing yak**	**Fattening yak**	***P***
L*_45min_	31.76 ± 4.39^A^	22.73 ± 3.52^B^	<0.01
a*_45min_	65.49 ± 3.83^A^	56.32 ± 4.18^B^	<0.01
b*_45min_	26.23 ± 3.72^a^	20.10 ± 3.05^b^	0.012
${\text{L}}_{24\text{h}}^{*}$	32.18 ± 4.24	28.31 ± 4.63	0.164
a*_24h_	73.01 ± 7.52^a^	65.22 ± 4.13^b^	0.049
b*_24h_	32.93 ± 6.33	29.18 ± 5.16	0.291
PH_45min_	5.93 ± 0.55	6.29 ± 0.35	0.212
PH_24h_	5.35 ± 0.33	5.22 ± 0.26	0.465
Drop loss (%)	20.36 ± 0.38^A^	18.11 ± 0.62^B^	<0.01
Shear force(N)	10.88 ± 1.55^a^	8.76 ± 1.00^b^	0.026
Conductivity(S·m^−1^)	2.71 ± 0.42	2.65 ± 0.29	0.804
Cooking loss (%)	37.21 ± 3.33^A^	29.93 ± 1.10^B^	<0.01

### Composition of Meat Chemical

**Figure 1:**
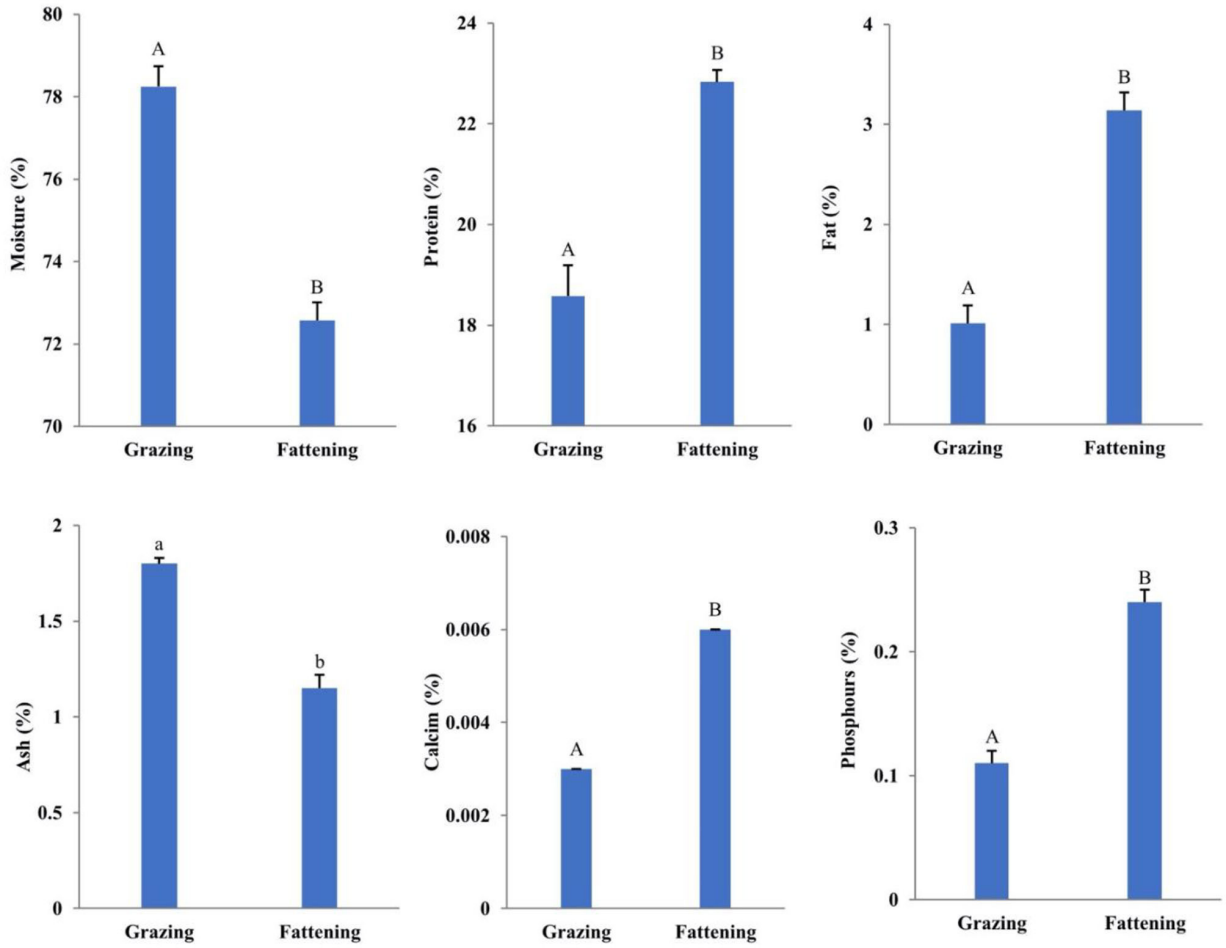
Effects of intensive fattening with TMR on the content of moisture, protein, fat, ash, calcium, and phosphorus in LD of yaks. The six figures in Figure 1, respectively, show the contents of moisture, protein, fat, ash, calcium, and phosphorus in the LD nutrients of grazing and fattening yaks (%). In the same group, a and b represent significant differences (*P* < 0.05), while A and B represent extremely significant differences (*P* < 0.01). The data are all the actual test values.

Figure 1 depicts the result of conventional nutritional components of LD in yaks under two feeding patterns. The observed results showed that the crude protein content, water content, fat content, including mineral elements of calcium and phosphorus in fattening yak muscle, have significant differences (*P* < 0.01), while the ash content was not very significant (*P* < 0.05).

Table 4 presents the fatty acid profiles of the LD of yaks. A total of 31 and 32 fatty acids were detected in grazing and flattening yak muscles, respectively. It was not possible to detect three fatty acids, trans-C_18:2_, C_20:1n9_, and C_21:0_ in grazing yak muscle. Two fatty acids, C_11:0_ and C_22:6N3_ were not detected in the fattening yak muscle. Besides, three fatty acids of C_22:1_, C_20:3N3_, and C_22:2_ were not detected in either of these two muscles. The most abundant fatty acids in the grazing yak muscle were found to be C_16:0_, C_18:0_, Cis-C_18:1_, CIS-C_18:2_, and C_20:4N6_. These five fatty acids accounted for 83.95% of the total fatty acids. The most abundant fatty acids in the fattening yak muscle came as C_16:0_, C_18:0_, CIS-C_18:1_, CIS-C_18:2_, and C_20:4N6_, concerning 88.01% of the total fatty acids. While fattening had little effect on SFA and UFA content in the yak muscle (*P* > 0.05), PUFA and MUFA content had significantly changed (*P* < 0.05). PUFA/SFA has shown a considerable difference (*P* > 0.05) as the proportion of polyunsaturated fatty acids has decreased. However, MUFA/PUFA content has also changed (*P* < 0.05) as the proportion of PUFA in UFA has been reduced.

**Table 4:** Effects of the intensive fattening with TMR on the fatty acid composition in *longissimus dorsi muscle* of yaks (mean ± SD, g/100 g).

**Fat acid**	**Grazing yak**	**Fattening yak**	***P***
C4:0	0.84 ± 0.18^A^	0.33 ± 0.13^B^	<0.01
C6:0	0.09 ± 0.02^A^	0.02 ± 0.01^B^	<0.01
C8:0	0.06 ± 0.02^a^	0.03 ± 0.01^b^	0.042
C10:0	0.15 ± 0.05	0.13 ± 0.02	0.397
C11:0	0.03 ± 0.01	ND	
C12:0	0.17 ± 0.03^A^	0.09 ± 0.02^B^	<0.01
C13:0	0.07 ± 0.02	0.04 ± 0.03	0.108
C14:0	1.77 ± 0.42	2.44 ± 0.36	0.011
C14:1	0.25 ± 0.04^A^	0.40 ± 0.08^B^	<0.01
C15:0	0.62 ± 0.18	0.33 ± 0.09	0.014
C15:1	0.36 ± 0.11	0.18 ± 0.07	0.012
C16:0	20.96 ± 0.41^A^	25.11 ± 1.20^B^	<0.01
C16:1	2.61 ± 0.74^A^	4.28 ± 0.69^B^	<0.01
C17:0	1.66 ± 0.59^a^	0.88 ± 0.18^b^	0.022
C17:1	1.41 ± 0.17^A^	0.76 ± 0.15^B^	<0.001
C18:0	21.13 ± 0.92	19.12 ± 2.37	0.112
*trans*-C18:1	0.53 ± 0.21^a^	0.97 ± 0.29^b^	0.016
*cis-*C18:1	16.88 ± 0.23^A^	28.48 ± 5.17^B^	<0.01
*trans*-C18:2	ND	0.14 ± 0.06	
*cis*-C18:2	10.72 ± 0.97	10.00 ± 2.46	0.533
C20:0	0.47 ± 0.08^A^	0.12 ± 0.02^B^	**<**0.01
C18:3n6	0.29 ± 0.05^A^	0.11 ± 0.07^B^	<0.01
C20:1n9	ND	0.20 ± 0.07	<0.01
C18:3n3	1.38 ± 0.21	0.79 ± 0.33	<0.01
C21:0	ND	0.10 ± 0.04	
C20:2	0.26 ± 0.06^A^	0.04 ± 0.03^B^	<0.01
C22:0	0.09 ± 0.03^A^	0.02 ± 0.01^B^	<0.01
C20:3n6	1.65 ± 0.26^A^	0.31 ± 0.15^B^	<0.01
C22:1	ND	ND	
C20:3n3	ND	ND	
C20:4n6	11.14 ± 0.45^A^	3.29 ± 0.63^B^	<0.01
C23:0	0.66 ± 0.15^A^	0.01 ± 0.00^B^	<0.01
C22:2	ND	ND	
C24:0	1.75 ± 0.31^A^	0.86 ± 0.45^B^	<0.01
C20:5n3	1.70 ± 0.26^A^	0.32 ± 0.11^B^	<0.01
C24:1	0.25 ± 0.07^A^	0.08 ± 0.05^B^	<0.01
C22:6n3	0.05 ± 0.00	ND	
SFA	52.38 ± 1.81	51.47 ± 0.67	0.808
PUFA	25.20 ± 1.51^A^	13.52 ± 1.65^B^	<0.01
MUFA	22.37 ± 1.94^a^	34.99 ± 1.15^b^	0.042
UFA	47.57 ± 1.17	48.51 ± 0.81	0.808
SFA/UFA	1.10 ± 0.39	1.06 ± 0.16	0.591
n-3 PUFA	0.76 ± 0.32	0.76 ± 0.33	0.423
n-6 PUFA	10.96 ± 0.45	3.32 ± 0.10	0.022
n-6/n-3 PUFA	14.41 ± 0.35	4.37 ± 0.26	<0.01

*ND stands for undetected, and the minimum detection limit is 0.0033%; The unit of fatty acid content was % and the ratio unit was 1*.

Table 5 presents the composition of amino acids in the LD of yaks. In the present study, a total of 16 amino acids were detected in fattening and grazing yak muscles, including phenylalanine (Phe), alanine (Ala), methionine (Met), proline (Pro), glycine (Gly), glutamic (Glu), arginine (Arg), lysine (Lys), tyrosine (Tyr), leucine (Leu), serine (Ser), threonine (Thr), asparagine (Asp), valine (Val), isoleucine (Ile), and histidine (His). Most of the amino acids mentioned above are higher in the flattening yak muscle than in the grazing group (*P* < 0.05). However, the level of Met in the muscle was not much different between grazing yak and fattening yak (*P* > 0.05).

**Table 5:** Effects of the intensive fattening with TMR on the amino acid composition in *longissimus dorsi muscle* of yaks (mean ± SD, g/100 g).

**Amino acid**	**Grazing yak**	**Fattening yak**	***P***
Phe	0.87 ± 0.04^A^	1.17 ± 0.04^B^	<0.01
Ala	1.19 ± 0.05^A^	1.60 ± 0.05^B^	<0.01
Met	0.32 ± 0.06	0.52 ± 0.13	0.11
Pro	0.68 ± 0.01^A^	0.88 ± 0.02^B^	<0.01
Gly	0.89 ± 0.01^A^	1.14 ± 0.02^B^	<0.01
Glu	3.18 ± 0.13^A^	4.24 ± 0.18^B^	<0.01
Arg	1.34 ± 0.04^A^	1.78 ± 0.07^B^	<0.01
Lys	1.94 ± 0.09^A^	2.61 ± 0.11^B^	<0.01
Tyr	0.72 ± 0.03^a^	0.97 ± 0.06^b^	0.01
Leu	1.71 ± 0.08^A^	2.33 ± 0.09^B^	<0.01
Ser	0.83 ± 0.02^A^	1.08 ± 0.04^B^	<0.01
Thr	0.98 ± 0.04^A^	1.31 ± 0.05^B^	<0.01
Asn	1.90 ± 0.08^A^	2.55 ± 0.10^B^	<0.01
Val	0.98 ± 0.04^A^	1.32 ± 0.05^B^	<0.01
Ile	0.92 ± 0.04^A^	1.31 ± 0.08^B^	<0.01
His	0.88 ± 0.07^a^	1.23 ± 0.06^b^	0.01
TAA	19.33 ± 0.032^A^	26.04 ± 0.042^B^	<0.01
EAA	8.60 ± 0.53^a^	11.80 ± 0.62^b^	0.02
NEAA	10.73 ± 0.46^a^	14.25 ± 0.65^b^	0.02

*Total amino acid (TAA), Essential amino acid (EAA) and Nonessential amino acid (NEAA) were the statistical result of the actual detection value in this experiment*.

### Serum Profile and Transcriptomic Profiles

The GLU, ALB, TRIG, and AMYL values in the fattening yak serum were considerably higher than those in the grazing yak (*P* < 0.05). Besides, the CREA value in the fattening yak serum was also higher than that in the grazing yak (*P* < 0.01). Subsequently, the values of LIPA and CK, LDH in the fattening yak serum were decreased (*P* < 0.05 and *P* < 0.01), respectively. There were no differences in ALT, ALKP, CHOL, and LAC in serum between intensive fattening yak and grazing yak (*P* > 0.05) (Table 6).

**Table 6:** Effects of the intensive fattening with TMR on blood biochemical indexes of yak (mean ± SD).

**Items**	**Grazing yak**	**Fattening yak**	***P***
GLU (mmol/L)	4.08 ± 0.51^A^	5.83 ± 0.26^B^	<0.01
CREA (μmol/L)	75.83 ± 8.50^A^	112.33 ± 11.43^B^	<0.01
ALB (g/L)	25.00 ± 4.34^a^	33.67 ± 6.89^b^	0.036
ALT (U/L)	66.00 ± 10.83	39.67 ± 16.23	0.088
ALKP (U/L)	121.17 ± 49.75	85.33 ± 28.45	0.164
CHOL (mmol/L)	2.01 ± 0.61	1.33 ± 0.95	0.175
TRIG (mmol/L)	0.13 ± 0.03^a^	0.19 ± 0.02^b^	0.025
AMYL (U/L)	45.50 ± 8.60^A^	58.50 ± 5.01^B^	<0.01
LIPA (U/L)	55.33 ± 2.25^a^	51.83 ± 1.33^b^	0.018
CK (U/L)	971.67 ± 66.36^A^	199.83 ± 28.07^B^	<0.01
LAC (mmol/L)	6.95 ± 1.37	5.71 ± 0.94	0.073
LDH (U/L)	1479.83 ± 31.16^A^	984.33 ± 85.21^B^	<0.01

**Figure 2:**
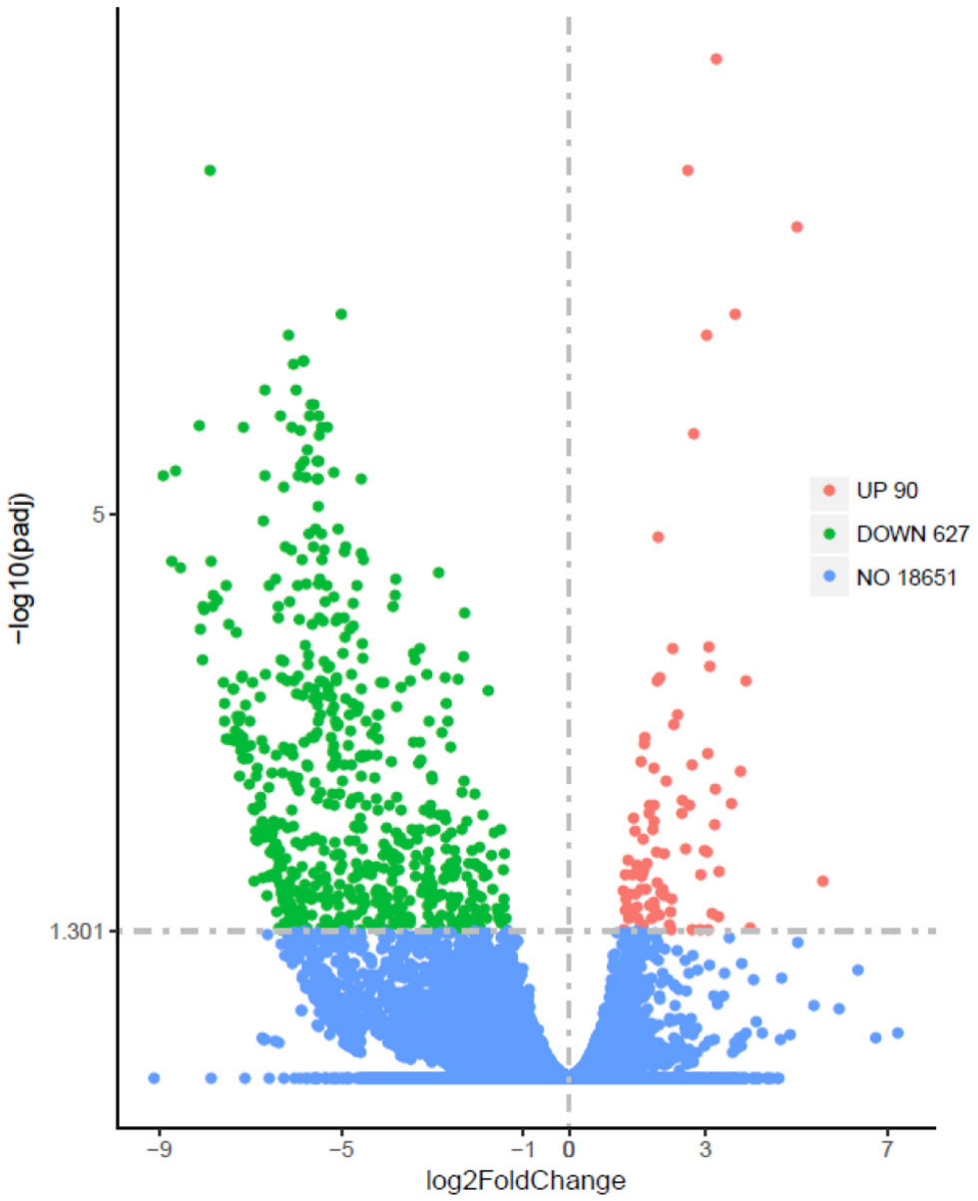
Differentially expressed genes (padj<0.05, llog2FoldChangl>O) between LD of fattening yaks and grazing yaks. Red denotes significantly up-regulated and green denotes down-regulated genes.

**Figure 3:**
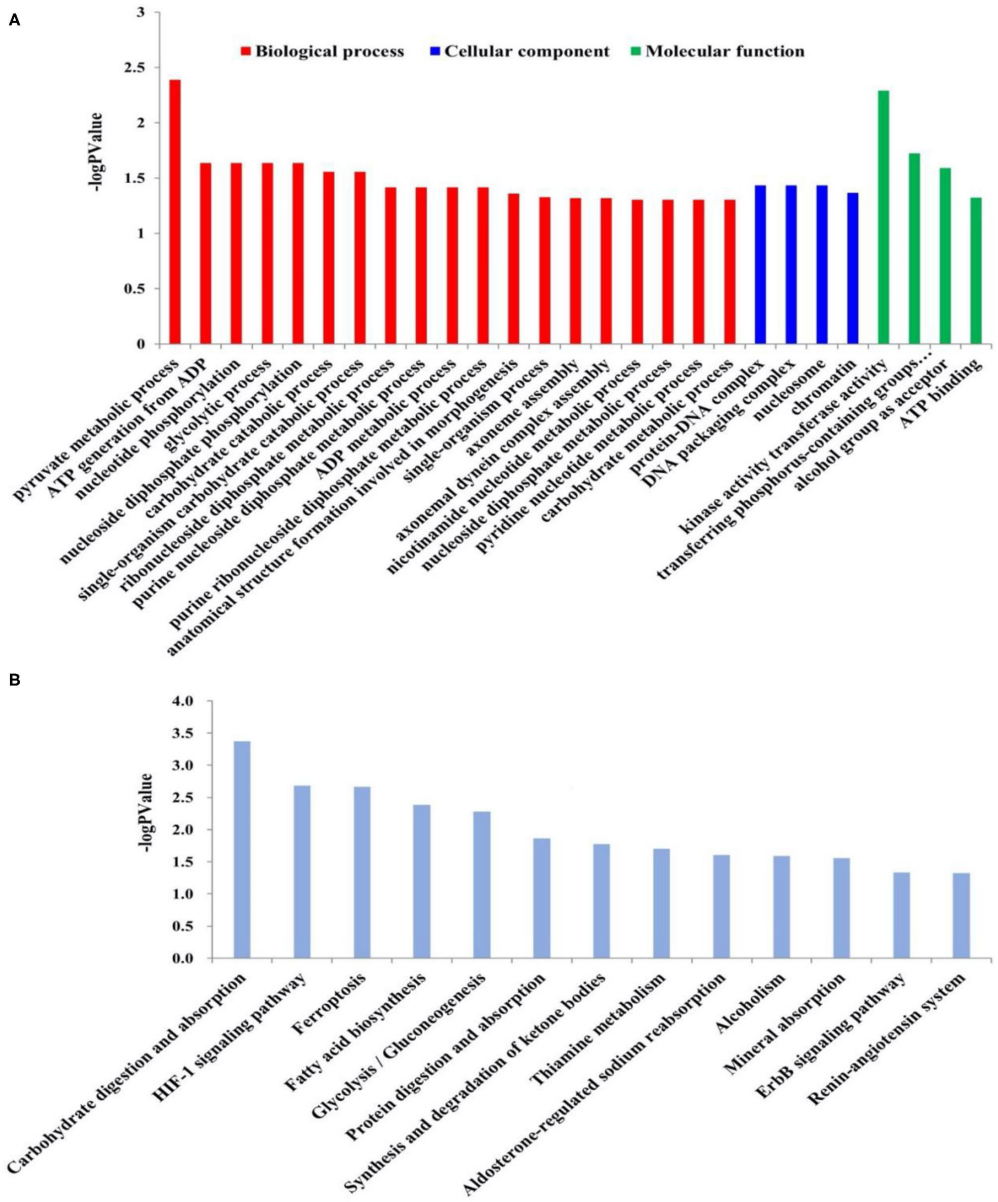
**(A)** GO enrichment analysis of the DEGs in LD of fattening yaks and grazing yaks. The GO terms belonging to biological processes, cellular components and molecular functions were shown in red, blue, and green, respectively. The significance levels were *P* < 0.05. **(B)** KEGG pathway analysis of the DEGs in LD of fattening yaks and grazing yaks. The significance levels were *P* < 0.05.

The Illumina sequence yielded 394,635,614 raw reads. A total of 191,212,296 clean reads were obtained for the fattening group with Q20 values of 98.06–98.17%, whereas 195,300,278 clean reads were received for the grazing group with Q20 values of 97.92–98.12%. A total of 717 DEGs, including 90 up-regulated and 627 down-regulated genes, have been identified between grazing yak and fattening yak, as shown in Figure 2. The expression values of the DEGs in each sample were analyzed by hierarchical clustering method. The obtained result confirms that the DEGs of the two groups may completely separate the grazing yak from the fattening yak, indicating that the expression differences of the DEGs in the two groups are significant. Enrichment analyses were carried out to investigate the functional association of the DEGs, GO, and KEGG. In the LD of yaks, 27 GO terms were significantly (*P* < 0.05) annotated within three major function groups, as shown in Figure 3A, also included the data in Table S1. Nine biological processes (e.g., pyruvate metabolic process, ATP generation from ADP, nucleotide phosphorylation, glycolytic process, nucleoside diphosphate phosphorylation, carbohydrate catabolic process, single-organism carbohydrate catabolic process) and four molecular functions (kinase activity transferase activity, transferring phosphorus-containing groups phosphotransferase activity, alcohol group as acceptor and ATP binding) have been included with 11 GO terms, in which nine genes have been identified with TMR effect on the growth mechanism of fattened yaks. Among them, four showed positive selection during the fattening yak growth. In these, the proportion of serine enzyme activity was found to be relatively high (26.77%), whereas the proteolysis and calcium ion binding were 20.11 and 5.58%, respectively. As shown in Figure 3B, 13 KEGG pathways with statistical significance were enriched based on the KEGG pathway enrichment analysis (*P* < 0.05), as presented in Table S2. There are seven genes related to the effect of TMR on the growth mechanism of fattening yak in five pathways. In these, the proportion is relatively high in digestion and absorption of carbohydrates (3.80%), glycolysis/gluconeogenesis (3.16%), and mineral absorption (2.53%).

## Discussion

### Effects of Fattening on Carcass Characteristics of Yak

Growth and development are important stages in animal life; fattening is a vital production goal for feeding animals, nutrition can have an impact on beef cattle losses, based on growth and development (23). Dietary nutrients eaten by beef cattle first meet the nutrient loss necessary to maintain normal life activities during their survival, and excess may be used for growth and weight gain. Therefore, an increase in the nutrient level of dietary intake can increase the nutrients used by beef cattle for growth and weight gain (9). In this experiment, the precisely matched TMR provides higher energy for yak, which maintains normal physiological life activities of the body and then converting into muscle and fat. The live weight and carcass weight are essential indexes for measuring the performance of meat production. Some parts of the matter comprising the grazing yak body were decomposed and used to maintain normal life activities during the cold season. In essence, from an economic point of view, the intensive fattening with TMR has a remarkable impact on the carcass characteristics, which can significantly improve the meat productivity of yak.

### Effects of Fattening on Meat Quality and Nutritional Value of Yak

The tenderness of meat is an important indicator of the edible quality of meat products; it is also an important factor for consumers to judge the meat quality and affect consumption. The tenderness was mainly determined by the content and distribution of connective tissue in the meat, the diameter of muscle fibers, sarcoplasmic protein content, and intramuscular fat content in the meat (17). The long-term grazing and raising of yaks and the aging of slaughtering lead to a poor tenderness of yak meat, which restricts its market acceptance (21). Many studies have shown that fattening can increase the intramuscular fat content in LD of yak, reduce shear force, and improve meat quality (2). In this study, the sheer force of LD for fattening yaks was significantly lower than that for grazing yaks as TMR promotes the synthesis and deposition of fat and protein between muscles.

The results showed that the fattening yak muscle's common nutritional value was better than the grazing yak muscle. Besides, the protein content, fat content, calcium content, and phosphorus content of fattening yak muscle were higher than the grazing yak muscle. However, the moisture content of meat decides the drop loss. Thus, lower moisture content of fattening yak muscle led to a lower drop loss and cooking loss. Simultaneously, researchers have shown that several factors control muscle tissue's moisture content; individual animal factors, protein content, fat content, pH, and pre-slaughter management control the moisture content (24). In this study, the protein content increased by fattening and inter-muscle fat content of the yak. Thus, with the increase in fat content and protein hydration, a certain amount of water is absorbed, making the inter-muscle water content of fattening yak lower than grazing yak.

The fatty acid composition of carcass affects not only the economic value of the carcass but also directly affects the health of consumers (15). Increasing awareness of the nutritional functions of PUFA has enhanced the studies on PUFA products, with a focus on eating meat with a high PUFA content and low content of UFA through modern healthy eating habits. Both PUFA/SFA and n-6/ n-3 are critical indicators to measure the nutritional value of fatty acids in the diet. WHO recommends that PUF/ SFA should be higher than 0.4, while the Chinese Society of Nutrition suggests that the n-6/ n-3 ratio must be in the range of 4-6 (5). Studies have shown that the higher the contents of N-6 and N-3 in natural grazing yak, the closer the ratio is to the recommended value. In the present study, the PUFA of the grazing yaks was 0.48, as it reached the recommended standard, whereas the PUFA of the fattened yaks was found to be 0.26; however, there is still a gap with the recommended value as fattening reduces PUFA content and increases MUFA content. While fattening sacrifices rich, healthy fatty acids in some muscles, it increases the total UFA content by improving its production and meat quality.

The content and composition of amino acids are the primary factors for determining protein quality. According to FAO/WHO standards, the proportion of high-quality proteins in EAA/TAA is ~40%, whereas EAA/ NEAA is close to 60% (5). In this experiment, EAA/TAA and EAA/NEAA in LD of grazing yak and fattening yak were also up to standard, showing that yak meat is a high-quality protein source, and fattening can promote the deposition of EAA. The results show that fattening increased only amino acids in muscle without changing its composition, consistent with the results obtained by others (2). The comprehensive analysis showed that the AAS score of fattening was greater, and phenylalanine (Phe) was considerably higher than that of the grazing yaks, indicating that fattening yaks' amino acid composition was better than grazing yaks, and thus the protein quality was better.

### The Mechanism Based on Profiles of Serum and Transcriptomic Profiles

Considering that yaks are ruminants, the effects of rumen fermentation, serum, and its metabolites on blood indexes have not been taken into account in this study; it is considered only from the perspective of yak growth and nutritional development, and energy metabolism. Thus, more nutrients, including fat, protein, and sugar in TMR, can raise GLU in serum. Due to lack and poor quality of forage during the cold season lead to nutrient deficiency, GLU in grazing yak (4.08 mmol/L) was lower than fattening yak (51.83 mmol/L). Thus, lactic acid, glycerinum, etc., were transferred into glucose to maintain normal blood sugar levels by glycolysis (bom00010). The grazing yak is needed to decompose fat in the body, and lipase (LIPA) is required for catalysis during lipolysis (25). LIPA in fattening yak (51.83 U/L) was lower than grazing yak (55.33 U/L). Here, a part of glucose in the body of fattening yak was used to generate energy catabolism, and this energy was used to sustain the life of yak. Excess glucose was transferred to fat and stored in the yak body, while triglycerides (TRIG) was increased during fat deposition. TRIG are the main components of fats, and the TRIG synthesis is affected by the acyl-CoA synthetase of long-chain family member 1 (ACSL1) and acetyl-CoA carboxylase beta (ACACB) enzyme (26). Plenty of TRIGs was synthesized by the high expression of ACSL1 and ACACB genes in fattening yak and deposited in their muscle. TRIG (0.19 mmol/L) in fattening yak serum was higher than grazing yak serum (0.13 mmol/L), reflect a high level of fat synthesis in fattening yak (bom00061). Expression of genes solute carrier family 7 member 8 (SLC7A8), sodium/potassium-transporting ATPase subunit alpha (ATP1A4), sodium/potassium-transporting ATPase subunit alpha-1 (ATP1A1), 4F2 cell-surface antigen heavy chain (SLC3A2), and the mast cell carboxypeptidase A (CPA3) improved the conversion efficiency of protein intake from TMR by weakening muscle proteolysis, enhancing amino acid absorption and self-proteins synthesis (27, 28). These synthetic proteins have been used for muscle growth. Thus, the protein content of a fattening yak muscle was higher than the grazing yak muscle.

Serum ALB plays a vital role in the physiological function of macromolecules *in vivo* and maintains nutritional levels in organisms, leading to lower ALB levels for causing malnutrition. Therefore, ALB was higher in fattening yak (33.67 g/L) than in grazing yak (25.00 g/L). In the cold season, grazing yaks suffered from malnutrition, which can be reflected by the lower ALB (25.00 g/L) in serum. In this situation, the yak living system had a kind of self-regulation to maintain normal life activities with the hope of obtaining more carbohydrates *in vitro*. Some genes ATP1A4 (29), ATP1A1 (24, 30, 31) play an essential part in the decomposition of glucose oxidation. The high expression of ATP1A4 and low expression of ATP1A1 can strengthen the process of ADP to ATP. Thus, carbohydrate digestion and absorption (bom04973) in grazing yak has been enhanced. The high CK level in serum generally indicates muscle damage. The grazing yak receives part of the energy from sarcolysis to support the activity of life during the cold season. Thus, the CK in fattening yak (199.83 U/L) was lower than the grazing yak. Malnutrition may also lead to high LDH (32) in grazing yak. LDH is one of the vital gluconeogenesis enzymes; the activity of LDH depends on the energy requirement of cells (14). LDH in grazing yak serum was 1479.83 U/L, which was higher than 984.33 U/L found in fattening yak serum. Low expression of Glyceraldehyde-3-phosphate dehydrogenase, testis-specific (GAPDHS), phosphoglycerate kinase 2 (PGK2), and hexokinase 3 (HK3) limited the gluconeogenesis in fattening yak, which can prevent the expenditure of protein and fat and accelerate the growth of yak muscle. The AMYL in fattening yak serum was found as 58.50 U/L, which is higher than 45.50 U/L found in grazing yak. Compared with forage and TMR, standard diets, especially starch sources, are used to finish beef cattle rich in concentration to promote high daily gains (33). Starches need to be degraded under the synergistic action of AMYL and phosphorylase (34), which also leads to fattening yaks AMYL higher than grazing yaks. This result shows that the fattening yak could get more glucose by the amylohydrolysis with AMYL as catalysts; thus, fattening yak could get enough nutrition by TMR.

HK3 enzyme can phosphorylate fructose to fructose 6-phosphate that participates in the glycolysis process (35). High expression of Pyruvate dehydrogenase E1 component subunit alpha (PDHA2) catalyzes the oxidative decarboxylation of pyruvate through gluconeogenesis (30), GAPDHS enzyme can catalyze glucose formation from glucose 6-phosphate (31), Fructose-1,6-bisphosphatase 1 (FBP1) regulates the activity of several enzymes in gluconeogenesis and intensified glycolysis (bom00010) in grazing yak, in particular the activation of phosphofructose kinase. KEGG pathways followed the absorption of minerals (bom04978) in fattening yak. Due to the difference in alkaline phosphatase (ALPL) expression, the fattening yak can obtain more calcium and phosphorus from TMR compared with grazing yak. The mineral absorption has increased in fattening yak so that more mineral substances, including calcium and phosphorus, allows yak to absorb more calcium, phosphorus and other minerals from TMR.

## Conclusions

The effects of intensive fattening with TMR on the carcass characteristics, meat quality, and the chemical composition of meat from yak have been reported in this paper. Based on blood biochemical and transcriptomic profiles of blood, the TMR nutrients are well-transformed into yak muscle nutrition, promoting the synthesis and deposition of yak muscle fat and protein. In the cold season, adopting the TMR-enhanced fattening feeding mode has a significant impact. It not only improves the weight loss of yaks, but the meat quality is also improved. Besides, from the perspective of nutritional value, fattening yak meat serves more healthy meat products that meet the demand of consumers.

## Data Availability Statement

The datasets generated for this study can be found in NCBI BioProject, NCBI Accession No. PRJNA631590.

## Ethics Statement

The animal study was reviewed and approved by Animal Administration and Ethics Committee of Lanzhou Institute of Husbandry and Pharmaceutical Sciences of Chinese Academy of Agricultural Sciences (Permit No. 2019-002).

## Author Contributions

PY and Q-LY conceived and designed the experiments and explained the data. The authors performed the experiments with the help of LX, X-MM, X-YW, and C-NL and analyzed the main content of the data. The authors wrote the manuscript with the help of PY and Q-LY. All authors contributed to the article and approved the submitted version.

## Conflict of Interest

The authors declare that the research was conducted in the absence of any commercial or financial relationships that could be construed as a potential conflict of interest.
